# Longitudinal Evaluation from Birth to Adolescence of Soy Protein–Based Infant Formula Compared with Cow Milk–Based Formula and Breastfeeding: A Comprehensive Summary of Findings

**DOI:** 10.1016/j.advnut.2026.100669

**Published:** 2026-06-06

**Authors:** Larissa L da Cruz, Julie Shlisky, B Jayne Bellando, Jin-Ran Chen, Tim Edwards, Chelsey Fiecke, Linda J Larson-Prior, Lana McCorkle, Mary Beth Moore, Chayla Slaton, Sarah Sobik, Keshari Thakali, Mara Whiteside, Aline Andres

**Affiliations:** 1 Arkansas Children’s Nutrition Center, Little Rock, AR, United States; 2 Department of Pediatrics, University of Arkansas for Medical Sciences, Little Rock, AR, United States; 3 Virginia Tech, College of Agriculture and Life Sciences, Blacksburg, VA, United States; 4 Department of Neuroscience, University of Arkansas for Medical Sciences, Little Rock, AR, United States; 5 Department of Radiology and Pediatrics, University of Arkansas for Medical Sciences, Little Rock, AR, United States

**Keywords:** soy-based infant formula, breastfeeding, infant feeding, children, adolescents, health outcomes

## Abstract

Breastfeeding is widely recognized as the gold standard food for infants. However, many families use infant formulas, including soy-based products, which have not been studied for their long-term safety and developmental effects. The Beginnings Study and the Beginnings Follow-Up Study represent one of the most comprehensive prospective cohorts designed to examine how early infant feeding is related to growth, body composition, cardiovascular, microbiome, and skeletal outcomes, neurodevelopment, and reproductive maturation through adolescence. Conducted in Arkansas, United States, the study enrolled 600 healthy, term infants fed soy-based infant formula, cow milk–based infant formula, or human milk during infancy (200 per group). Of these, 385 participants (73.2%) completed the 6-year visit, and 190 (31.7% of enrolled and 49.4% of 6-year visit completers) participated in the 14-year Follow-Up Study. Breastfeeding was associated with slower weight gain velocity during infancy, and consistent lower BMI, fat mass index, and waist circumference extending into adolescence, than formula feeding. Formula-fed infants had comparable results with breastfed infants for skeletal mineralization, most neurocognitive parameters, and reproductive organ development. However, cardiovascular autonomic measures, including heart rate and vagal tone, differed by feeding group, with some sex-specific effects. Novel contributions included analyses of the gut microbiome and metabolomics profiles in early life, which revealed distinct dietary signatures, as well as neurodevelopmental assessments using electroencephalography, which highlighted transient differences in language-related brain responses among feeding groups. Together, these results demonstrate more similarities than differences between soy-based infant formula and cow milk–based infant formula in health outcomes and support the lasting benefits of breastfeeding. This evidence can help guide health care professionals in infant feeding recommendations and highlight critical windows to prevent obesity and promote lifelong health.

**Statement of significance**

This landmark longitudinal cohort demonstrates that while breastfeeding is associated with consistent advantages in adiposity and cognitive function compared with formula feeding, soy milk– and cow milk–based infant formulas yield largely comparable outcomes between formula types across growth, neurodevelopment, skeletal health, and reproductive outcomes—providing critical evidence to inform infant feeding practices and identify early-life windows for lifelong health.

## Introduction

It is well established that breastfeeding can affect human health from infancy into adulthood, providing protection from acute and chronic diseases [1] such as obesity [2], type 2 diabetes [3], cardiovascular disease [4], and certain cancers in later life [5,6]. At the population level, these benefits translate into reduced morbidity and mortality and substantial savings in health care costs and productivity losses [7]. When breastfeeding is not possible, cow milk–based infant formula (MF) is recommended, which when not tolerated, use of soy-based infant formula (SF) is the recommended alternative [8].

SF is a plant-based formula designed as an option, due to clinical conditions, such as cow milk allergy (CMA), lactose intolerance, or parental dietary preferences [9]. The global SF market was estimated at United States $312.5 million in 2024, with projected growth to United States $431.0 million by 2032 [10]. SF has been used for several decades [11], although its consumption shows regional variation [12].

Worldwide SF use is difficult to estimate due to variation in its consumption among countries, partly due to the formulas types available, how formula use is prescribed and monitored, and limited data transparency within the formula industry [13]. However, available data show considerable variation among countries. SF accounts for ∼12% of infant formula use in the United States, which means that it is the second most consumed in this country [14]. In contrast, prescription-based data from England and Norway show that SF represents only a very small fraction of specialized formula use (<1%) [15]. In Asian countries, although population-level prevalence data remain scarce, SF is often used as a practical alternative for infants with CMA [16].

Unlike other infant feeding modalities, SF contains isoflavones, which are plant-derived compounds that are also referred to as phytoestrogens due to their chemical structure being close to that of estradiol [17]. Infants fed SF consume disproportionately high amounts of isoflavones relative to their body weight and at greater frequency than adults, leading to increased circulating concentrations of isoflavones that has been hypothesized to exceed metabolic capacity [12]. This distinct nutritional composition has raised safety concerns across multiple developmental outcomes [18]. Specifically, due to their structural similarity to estradiol, isoflavones, such as genistein and daidzein, can bind to estrogen receptors (ERs; ERα and ERβ) and act as agonists or antagonists depending on tissue and hormonal context [17]. This estrogenic activity has led to hypotheses regarding potential influences on reproductive and endocrine function as well as neurobehavioral development. Particular concern exists during the postnatal period known as minipuberty, since exposure to exogenous estrogenic compounds, such as soy isoflavones, during this sensitive window, may disrupt the programmed hormonal events through interference with normal hypothalamic–pituitary–gonadal axis [17]. SF also contains phytates, which are natural compounds that can bind minerals such as calcium. The presence of these nutritional factors such as mineral bioavailability have motivated the assessment of bone health [18,19].

Experimental studies in animal models have demonstrated that soy isoflavones can influence metabolic processes, and endocrine and immune systems during development, raising biologically plausible concerns, although species differences in pharmacokinetics and developmental timing limit extrapolation to humans [[20], [21], [22], [23], [24]]. Evidence from animal models studies suggest different pathways for these effects. High-isoflavone diets increased hepatic phase II enzyme activity, suggesting modulation of the metabolic detoxification pathways [20], while genistein could bind estrogen receptors and inhibit thyroid peroxidase, indicating potential endocrine-disrupting effects under iodine-deficient conditions [21]. Reproductive and immune systems can also be affected. In a multigenerational model, male offspring exposed to isoflavones at concentrations approximating SF intake showed elevated androgens [22], and maternal dietary exposure to daidzein and genistein suppressed cell-mediated immunity in male rat offspring, through reducing interferon γ and IL-12 concentrations [25]. While some animal studies raise concerns, the expert panel of the National Toxicology Program Center for the Evaluation of Risks to Human Reproduction concluded that SF poses minimal concern in humans, citing insufficient data for definitive conclusions [26].

Human data on the neurodevelopmental and endocrine effects of SF remain limited. Despite the elevated isoflavone exposure during hormonally sensitive developmental periods [17], evidence of SF influence on brain development and puberty remains inconsistent and is often disadvantaged by design limitations [12,27,28]. Retrospective and cohort studies report no consistent associations with adverse pubertal or neurodevelopmental outcomes or overall health, although women who consumed SF as infants reported slightly longer menstrual bleeding and more discomfort [[29], [30], [31]]. Despite decades of clinical use, critical gaps remain in the evidence base surrounding SF long-term safety and developmental outcomes remain incompletely characterized [14]. No longitudinal studies have tracked SF-fed infants through adolescence to assess associations with growth, cognition, cardiovascular and skeletal health, or reproductive development [17].

The impact of early SF exposure on the developing gut microbiome [19] and its downstream influence on neurodevelopment, immune programming, and metabolism are poorly understood [11]. Despite lacking preventive benefits for food allergy and potential delays in tolerance acquisition, SF continues to be widely used in healthy, nonallergic infants, often without clinical indication [12,32,33]. Existing human studies are limited by short follow-up, retrospective recall, and inadequate control for feeding exclusivity, and regulatory assessments continue to highlight the need for high-quality prospective data to confirm or rule out long-term risk [11,19].

**FIGURE 1:**
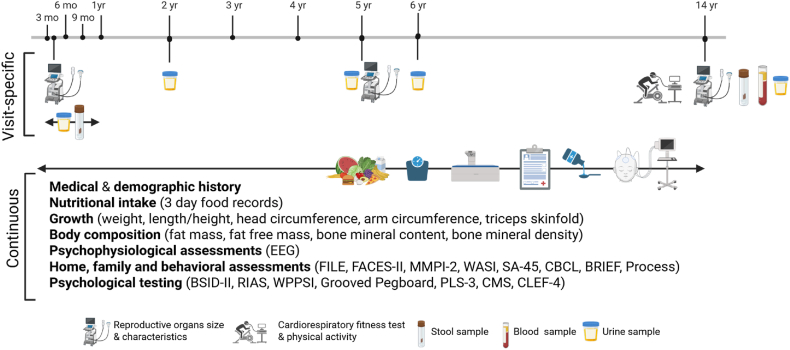
Beginnings Study and Beginnings Follow-Up Study data collection schedule. Data collection is categorized into visit-specific and continuous assessments. These categories and the study visit collections are defined according to the outcomes described in the Beginnings Study and the Beginnings Follow-Up Study protocols approved by the institutional review board. BRIEF, Behavior Rating Inventory of Executive Function; BSID-II, Bayley scales of infant development II; CBCL, Child Behavior Check List; CLEF-4, Clinical Evaluation of Language Fundamentals-Fourth Edition; CMS, Children’s Memory Scale; FACES-II, Family Adaptability and Cohesion Evaluation Scales-II; FILE, Family Inventory of Life Events and Changes; MMPI-2, Minnesota Multiphasic Personality Inventory-2 Questionnaire; PLS-3, preschool language scale-3; RIAS: Reynolds intellectual assessment scales; SA-45, Symptom Assessment 45; WASI, Wechsler Abbreviated Scale of Intelligence; WPPSI, Wechsler Preschool and Primary Scale of Intelligence.

With this background in mind, the Beginnings Study was a prospective, longitudinal investigation designed to evaluate how infant feeding—human milk (HM), MF, or SF—affects dietary intake, growth, body composition and skeletal health, metabolism, neurodevelopment, and reproductive outcomes from infancy through age 6 y. Six hundred healthy, full-term infants were enrolled based on parental feeding choice (exclusively HM, MF, or SF) during early infancy and repeated assessments across 10 study visits from 3 mo to 6 y. The Beginnings Follow-Up Study recalled these participants at age 14 to assess outcomes during adolescence (Figure 1). The study was designed to examine whether early exposure to soy isoflavones is associated with growth, body composition, cardiovascular, skeletal, reproductive, or pubertal outcomes, as well as microbiome composition and metabolomics profiles. To our knowledge, the Beginnings Study and its Follow-Up Study is the only United States prospective cohort to evaluate infant diet effects through adolescence, across anthropometric, biochemical, cognitive, dietary intake, and physiological domains. This extended follow-up provides a rare opportunity to assess whether early dietary exposures have delayed or re-emerging effects on BMI, cognitive performance, reproductive health, and cardiometabolic function.

## Methods

### Study designs

**FIGURE 2:**
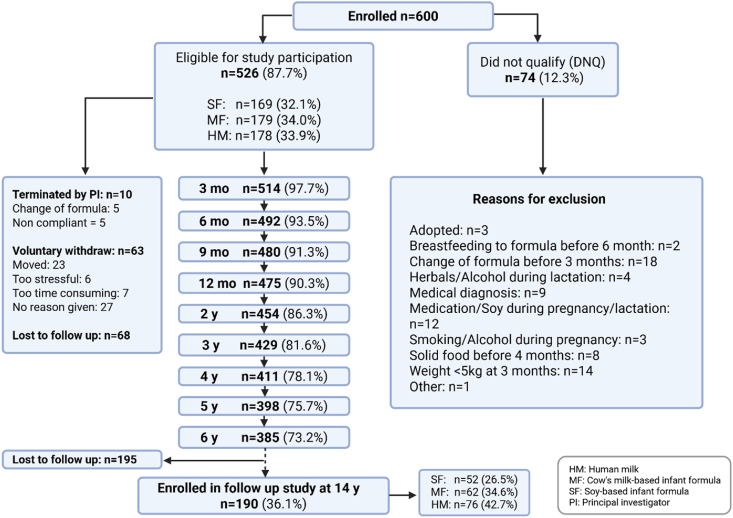
CONSORT flow diagram of participants through the Beginnings Study (3 mo to 6 y) and the Beginnings Follow-Up Study (14 y).

The Beginnings Study (clinicaltrials.gov identifier: NCT00616395) was a prospective study initiated at the Arkansas Children’s Nutrition Center in Little Rock, Arkansas, in 2002. It was designed to evaluate differences in physical growth, body composition, and neurodevelopment between infants and children who consumed SF, MF, or HM during the first year of life. Details related to the Beginnings Study design, inclusion and exclusion criteria, have been described previously [34]. Briefly, participants were recruited between 1 and 2 mo of age. Pregnancies were uncomplicated with no medical diagnoses or medications known to affect fetal or infant growth and development. Infants were term (>37 wk), between 2.7 and 4.1 kg at birth, had no known medical diagnoses, and had not been administered medications known to affect growth or development. Participants were excluded from the study if they were smokers, reported alcohol use during pregnancy or use of soy products or other potential estrogenic compounds during pregnancy and/or lactation, changed formula between 2 and 12 mo of age, or introduced complementary foods before 4 mo of age or if the infant body weight at 3 mo of age was <5 kg. Study visits were conducted at 3, 6, 9, and 12 mo and then every year until 6 y of age. Data were collected from 2002 to 2011. The Beginnings Study enrolled 600 children (*n* = 200 per feeding mode). Of these, 526 met eligibility criteria during the first year of life, and 385 participants (64.2% of enrolled; 73.2% of participants meeting eligibility criteria) completed the 6-y visit (Figure 2).

Prior participants from the Beginnings Study who had consented to be recalled for future studies were contacted for a follow-up study visit at 14 y (Beginnings Follow-Up Study; NCT03108014) to evaluate the associations between feeding modalities during infancy (SF, MF, or HM) and growth, development and sexual maturation. One hundred and ninety 14-y-old (range, 13.47–14.96 y) adolescents were enrolled and completed the study visit between 2017 and 2024 (Figure 2), including 52 children fed SF, 62 children fed MF, and 76 children fed HM during infancy. In this review, we are presenting the outcomes that have already been analyzed at 14 y old, such as anthropometrics and body composition, dietary intake and diet quality, reproductive organs, and sexual maturation. Other parameters collected, such as cognitive and behavioral assessments, physical activity, and skeletal outcomes, are under analysis and will be reported in future publications.

### Infant diets

Details related to infant feeding in the Beginnings Study have been reported previously [34]. Briefly, parents decided to feed their infants SF, MF, or HM, based on clinical considerations, prior experience, or personal preference prior to enrollment. Those who chose to feed formula were provided MF (Similac Advance or Enfamil Lipil) or soy-protein based formulas (Similac Soy Isomil or Enfamil Prosobee) to ensure standardization of infant dietary intake. The 2 brands were available to reflect the 2 most prevalent formulas on the market at the time of the study, which allowed most parents to continue to feed their children their formula of choice. Infants fed SF or MF remained on the selected formula until 12 mo of age. For infants fed HM, breastfeeding was encouraged until 12 mo of age, and 53% (*n* = 70) did. The remaining 47% (*n* = 61) were weaned between 6 and 12 mo: 10% (*n* = 13) were breastfed until 6 mo, 21% (*n* = 27) until 8 mo, and 6% (*n* = 8) until 10 mo, and 10% (*n* = 13) initiated mixed feeding after 6 mo and continued until 12 mo [35]. For HM-fed infants, mothers recorded the number of feeds per day and estimated average volume intake. Since HM composition varies through the day according to the children’s age, HM macronutrients were assumed to be 0.044 g/mL of fat, 0.069 g/mL of carbohydrates, and 0.010 g/mL of protein, based upon the standardized values derived from the Nutrition Data System for Research (NDSR) [36]. Complementary foods could be introduced after age 4 mo for all diet groups to align with American Academy of Pediatrics recommendations at the time of the study.

### Child dietary intake

Dietary information was collected for the child at each study visit using 3-d food records. During infancy and childhood, food records were completed by parents. At the 14-y visit, they were completed by the child with parental assistance, as needed. Food records were reviewed by a trained nutritionist with the participant and/or the parent to obtain detailed information for each meal. A daily mean of energy, macronutrients, and micronutrients intake were obtained through the NDSR software [Nutrition Coordinating Center (NCC)] in accordance with NCC recommendations [36].

### Physical development

#### Anthropometry

Anthropometric measurements (weight, length/height, head circumference, and waist circumference) were obtained using standardized techniques at each study visit. The specific measurements collected varied according to the participant’s age (eg, head circumference and length were measured in infants, while height was measured in children and adolescents). Weight-for-length *z*-scores were computed from the WHO growth charts using Anthro version 3.2.2. BMI (in kg/m^2^) was calculated for children (≥24 mo) [34].

#### Body composition and skeletal health

Body composition was assessed by dual-energy x-ray absorptiometry (QDR 4500 with Discovery upgrade; Hologic), with only artifact-free scans used for analysis (QDR software for Windows XP, version 12.3; Hologic). Bone mineral content (BMC), bone mineral density (BMD), fat mass (FM), and fat-free mass (FFM) were calculated using the QDR software. Fat mass index (FMI) and fat-free mass index were calculated as previously described [34]. Skeletal health was assessed using peripheral quantitative computed tomography on the nondominant radius, according to the manufacturer’s recommendations. Scans were acquired at 4% and 66% of the radial length; scans exhibiting motion artifacts were excluded from the analysis. Density thresholds and acquisition parameters were applied as previously described [37].

### Cardiovascular and nervous system

Resting heart rate, heart period, and vagal tone measures were obtained as described previously [38]. Briefly, recordings were obtained from bilateral precordial placements at V4 taken while infants were awake, in a light-dimmed, electrically isolated, and sound attenuating room. Heart rate recordings were digitized at 1000 Hz and screened; children with <90 s of acceptable data were not included. Heart period was calculated as the average of the R-R intervals across the rest period. Vagal tone was determined using the method developed by Porges [39] and calculated in sequential 30-s epochs, and the means of these epochs for the baseline period were used in analyses.

### Reproductive development

#### Pubertal status

Pubertal status was made by self-assessment, using the Sexual Maturity Rating [40,41], which evaluates pubic hair growth and external genitalia development for both sexes, and breast development in girls [42]. Girls were also asked about age of onset of menarche.

#### Sonograms

Sonogram measurements were performed at the Radiology Department of the Arkansas Children’s Hospital by registered diagnostic medical sonographers at age 4 mo, 5 y, and 14 y. Images were reviewed and measured by 2 board-certified pediatric radiologists (MBM and ACR), who were blinded to participant feeding group, as were the sonographers. All organs volumes (ovaries, prostate, and uterus) were calculated in triplicate from 3 orthogonal planes (sagittal, transverse, and anteroposterior); for paired organs (ovaries and testes), measurements were obtained on each side. All organ volumes were standardized by body weight and expressed as cubic millimeters per kilogram.

### Urinary purine metabolites

Urine samples were analyzed at 3 mo of age following standardized procedures for the analysis of purine metabolites, as described before [43]. Urinary creatinine and purine metabolites quantification was performed as described previously [44]. Select purines (inosine and hypoxanthine) were also quantified using commercial assays kits [45]. Urinary concentrations of metabolites were normalized to creatinine, which was measured using a colorimetric assay [46].

### Stool microbiome and metabolome

Fecal samples were collected either during the study visit or at home at 3, 6, 9, and 12 mo, and stored at −80°C until analysis. For microbiome analysis, DNA was extracted, and the hypervariable region 4 of bacterial 16S ribosomal RNA was amplified and sequenced, as described previously [38,39]. Sequence reads were analyzed using QIIME 1.9.1 [47]. Fecal samples (100 mg) were also subjected to untargeted metabolomics analyses [48].

### Neurodevelopmental outcomes

#### Child neurocognitive and language skills

Neuropsychological technicians, supervised by a licensed psychologist and blinded to the study groups, administered the Bayley Scales of Infant Development II between ages 3 and 24 mo to obtain the mental developmental index and psychomotor development index [49]. Maternal intelligence quotient was estimated at the 3-mo visit using the Wechsler Abbreviated Scale of Intelligence [50]. The child’s temperament was evaluated using the parent reported Carey Temperament Scales [51] at each visit between 6 mo and 6 y. All other neurodevelopmental measures occurred at the 6-y visit. The Wechsler Preschool and Primary Scale for Children—Third Edition [52], provided a measure of cognitive development, the Full-Scale Intelligence Quotient. The Preschool Language Scale-3 [53] provided comprehensive information about language development reported as total language score, auditory comprehension score, and expressive communication score. The Children’s Memory Scale [54] evaluated learning and memory functioning, which was reported using the general memory, delayed recognition, attention/ concentration, and learning index scores.

#### Neurophysiological measures

High-density (128 channel nets) electroencephalograms were collected at each study visit, under various paradigms, all of which were considered resting state electroencephalograms [55]. Data were preprocessed in MATLAB using the standard Harvard Automated Processing Pipeline for Electroencephalography [56] and re-referenced using the references electrode standardization technique [57], while data quality was evaluated using the Brainstorm software package [58]. Using a standardized infant brain atlas [59], boundary element head models were generated, and power spectral density was calculated using Welch method across canonical frequency bands (δ, θ, α, β, and γ) following previously described procedures [60].

### Covariate selection

For each analysis conducted, selection of covariates was determined by directed acyclic graphs, conceptual frameworks and prior evidence relevant for the specific outcome domain under investigation. The most common covariates used can be included into 4 categories: prenatal factors (e.g. gestational age, birth weight, birth length, and head circumference), parental and socioeconomic characteristics [61] (e.g. maternal and paternal education level, parental income, and maternal IQ), child characteristics (e.g. gender, race/ethnicity, anthropometrics, and body composition), and diet-related variables (e.g. energy intake, age at feeding group onset, and time of solid food introduction). Detailed covariates used for each specific outcome are presented in Supplemental Table 1.

In most analyses, length of breastfeeding was used as a covariate to account for mothers who discontinued breastfeeding after 6 mo and before 12 mo. In some cases, sensitivity analyses were conducted including only children who were breastfed for 12 mo to validate the results of the whole cohort [62]. All participants were transitioned to dairy-based infant formula if they discontinued breastfeeding. The study did not assess or control for breast pump use.

## Results and Discussion

### Overview and context

The Beginnings Study and the Beginnings Follow-Up Study provide a comprehensive, longitudinal evaluation of the associations between infant feeding and growth, body composition, dietary intake, cardiovascular, neurodevelopmental, gut, skeletal, and reproductive outcomes from infancy through adolescence (Figure 1). Dietary intake [34,63], skeletal health [37], and reproductive development parameters [[63], [64], [65]] were not different between feeding groups; however, significant differences were observed in body composition [34,63,66], cardiovascular parameters [67,68], microbiota profiles [69], and neurodevelopmental measures [35,62,[70], [71], [72], [73], [74], [75], [76], [77], [78], [79]].

When the Beginnings Study enrolled the first participants in 2002, ∼35% of infants in the United States were breastfed at 6 mo of age, with only 13% receiving exclusive breastfeeding [80]. Little evidence existed regarding the health implications of phytoestrogens found in SF, despite its widespread global use. Theoretical endocrine-disrupting effects, including possible associations with precocious puberty in girls and altered reproductive development in boys, had been proposed, largely based on in vitro and experimental findings [81]. Data to examine the health effects of widespread use of formula feeding, including SF feeding, were not available.

### Dietary intake and physical development

#### Nutrition

During the first year of life, energy, carbohydrate, and protein intake were significantly lower, whereas fat intake was significantly higher, for HM-fed infants than for formula-fed infant groups. Although caloric intake did not differ between the 2 formula groups, the distribution of macronutrients (protein, fat, and carbohydrates) differed between SF-fed and MF-fed groups, as described previously [34]. From 2 to 6 y, there were no differences in energy or macronutrient intake between the 3 feeding groups [34]. At 14 y of age, energy intake, macronutrient intake, and diet quality were comparable across groups, except for a lower fat intake (in percentage kilocalories) for participants fed SF and HM as infants than in those fed MF [63]. This suggests that while infant feeding practices influence childhood dietary patterns, differences tend to lessen as children age and family diet becomes dominant [[82], [83], [84]]. Additional analyses of dietary diversity and polyphenol intake are planned to provide further clarity whether infant feeding modality influences later dietary patterns.

#### Body composition

Infants fed HM showed lower weight gain velocity during the first year of life, which resulted in lower weight-for-length *z*-scores at 12 mo, than formula-fed infants [66]. These differences persisted into childhood [34]. Although all feeding groups achieved adequate growth, HM-fed children had significantly lower BMI *z*-scores from 2 to 6 y than SF-fed children, and there were no significant differences between SF-fed children and MF-fed children, as described previously [34]. Similarly, while whole body fat mass (%) was higher in HM-fed infants early in life (3 and 6 mo of age) [34,85], HM-fed children had significantly lower FMI from age 3 to 6 y than formula-fed children, with no differences between SF- and MF-fed children [34]. By age 14 y, adolescents in the HM group had significantly lower FMI and waist circumference than those who were fed MF as infants. The SF group presented intermediate values, with no significant differences between SF-fed and either HM-fed or MF-fed adolescents. FMI was used during childhood because it is height normalized, so it is considered more appropriate during growth than body fat percentage [86]. Analyses of BMI-for age *z*-scores from 6 to 14 y showed no differences among groups; however, a higher prevalence of overweight and obesity was observed in the MF-fed group by age 14 y (Supplemental Figure 1) [34,63]. These findings support a protective effect of breastfeeding against excess adiposity, which is in line with a recent study demonstrating a lower risk of being overweight at 2 to 3 y of age for infants exclusively breastfed for 12 mo or longer than formula-fed infants [83]. The results are also in line with a meta-analysis of 25 studies including >226,000 participants [87], demonstrating that children who were breastfed during infancy had a 22% lower risk of childhood obesity than never breastfed children. These differences may be partially attributed to variations in early nutrient intake, as breastfed infants have significantly lower intakes of energy, protein, fat, and carbohydrates than formula-fed infants [84].

#### Skeletal health

The most common parameters used to assess the bone health in pediatric population are the BMC and the BMD. While BMC provides information about total bone mineral content (in grams), BMD is related to concentration of minerals within a specific bone area (in grams per square centimeter), and it is calculated from BMC and bone area. Despite these measures are related, BMD can be influenced by skeletal growth during childhood [88].

In the Beginnings Study, HM-fed infants had significantly higher (BMC) at 3 mo of age than formula-fed infants. However, by age 9 and 12 mo, BMC was higher in formula-fed than in HM-fed infants [66]. These results align with earlier reports [82] of higher BMC in formula-fed infants than in breastfed infants at age 12 mo. Although differences in BMC were observed in early infancy, these differences did not persist beyond the first year of life, with no statistically significant group differences in BMC or (BMD) detected between feeding groups, as assessed using dual-energy x-ray absorptiometry and peripheral quantitative computed tomography through 6 y of age [37]. These early differences in BMC probably reflect differences in body size and growth, rather than differences in BMD. These results are somewhat similar to findings from Dorrepaal et al. [89], who showed no associations between infant feeding modality [exclusive breastfeeding, exclusive cow milk formula feeding (MF), or mixed feeding (HM+MF)] on BMD at 6 mo or 3 y of age. Taken together, these findings suggest that early differences in BMC are transient and do not result in long-term disparities in skeletal development through early childhood [90]. However, the long-term implication beyond mid-childhood remains unclear. Future longitudinal studies should evaluate the process of bone remodeling in adolescence, with a focus on potential epigenetic and microbiota-mediated pathways [91] via the gut–bone axis [92], which may influence skeletal health across the lifespan.

### Cardiovascular and nervous system

Heart rate period and the resting vagal tone are important markers to understand the determinants of cardiovascular autonomic regulation [38,93]. Changes in those markers provide important information about the development of cardiac autonomic regulation, cardiovascular health, and the vagal tone has also been associated with cognitive function and socioemotional behavior in infants and children [38,94]. The rates of change in heart period were variable during the first year of life among feeding groups and were sex specific, with significant differences in boys but not in girls [38]. At 6 mo of age, the rate of change in heart period was greater for HM-fed boys than other feeding groups. Yet, by the age of 1 and 2 y, the rate of change in heart period was greater for SF-fed boys than that for HM-fed boys. Similarly, resting vagal tone was significantly higher in SF-fed boys than in HM-fed boys [38,67]. Despite these group differences, both vagal tone and heart period increased across time for all feeding groups, and the stability of vagal tone assessed was higher in HM-fed than in formula-fed infants during the first 2 y of life [67]. Taken together, these findings suggest that SF-fed boys differ from HM-fed boys in cardiovascular and autonomic nervous system outcomes, indicating that sex and environmental factors like diet are important modulators of the early development of autonomic state control [95,96]. More recent investigations during early development confirm that baseline vagal tone increases from infancy to early childhood [[97], [98], [99], [100]]. Additionally, throughout early childhood, there is moderate to modest stability in measures of baseline vagal tone [100], substantiating the hypothesis that vagal tone can be influenced by environmental factors during infancy and early childhood [101]. The modulation of vagal tone by diet components represents a promising avenue through which infant nutrition may exert long-lasting effects on health and behavior, although further clinical studies are needed to confirm the persistence, functional significance, and mechanisms of these early autonomic differences [102]. Future studies are warranted to investigate how environmental exposures, such as infant feeding modalities and maternal factors like diet and body composition, affect the association between autonomic nervous system development and behavioral and cardiovascular disease risk outcomes later in life.

### Reproductive development

At age 4 mo, MF-fed girls had higher ovarian volume and a higher number of ovarian cysts per ovary than HM-fed girls. No differences were observed between SF and HM or SF and MF groups. No other group differences were observed in girls for breast bud, uterine, or ovarian characteristics. The ovarian volume and the ovarian cysts are parameters used to characterize ovaries across groups [103]. For boys, HM group had higher testicular volumes at 4 mo than formula-fed infants [64]. No other group differences were observed in boys for breast bud or prostate characteristics. By age 5 y, no differences were observed between feeding groups for the volumes and characteristics of breast buds, uterus, ovaries, testes, or prostate [65]. At age 14 y, there were also no differences in reproductive organs (ovaries and uterus) sizes or characteristics, age of menarche, or sexual maturity rating in girls between feeding groups. Similarly, at age 14 y, there were no differences in reproductive organ (breast buds, prostate, and testes) size and position (testes) or sexual maturity rating in boys between feeding groups [63].

While the literature suggests that isoflavones may impair reproductive function in experimental models [104], there is a dearth of evidence on the effect of SF on reproductive organ volumes, structural characteristics, and pubertal development in children [29,105]. To our knowledge, the Beginnings Study is the first prospective evaluation of SF feeding on reproductive organ volume as participants underwent ultrasound assessments of their reproductive organs at age 4 mo, 5 y, and 14 y with pubertal development evaluation at 14 y. No long-term differences were observed across feeding groups in reproductive organ size or pubertal development [63]. The lack of adverse reproductive effects among SF-fed participants until 14 y old supports existing studies that suggests that SF use is not associated with relevant reproductive development in children and adolescents [12]; however, the long-term effects of phytoestrogen exposure on reproductive outcomes throughout their reproductive years cannot be definitely evaluated, and further investigation are needed. A recent narrative review [106] noted potential associations between longer breastfeeding duration and slightly delayed pubertal onset in boys. However, findings across studies are inconsistent and were not observed in the Beginnings Study. Overall, these results suggest that SF is not associated with differences in reproductive organ sizes, reproductive organ characteristics, or pubertal development between ages 4 mo and 14 y compared with MF or HM [[63], [64], [65]].

### Urinary metabolome

Two analyses revealed that urine samples from SF-fed infants were associated with a distinct urinary metabolome using untargeted GC–time-of-flight MS during the first year of life [43,107]. The first analysis revealed significantly higher urinary purine pathway outputs (13 different purine metabolites) in SF-fed infants using targeted assays at 3 mo of age than MF- or HM-fed infants [43]. These results align with the sparse infant purine-specific literature, showing that higher soy-formula purine intake increases urinary uric acid excretion and with official composition syntheses reporting substantially higher nucleotide content in soy protein–based formulas than in HM or MF [108]. Using an untargeted approach, the second analysis found 150 metabolites whose abundance differed between feeding groups. Infants who were fed HM had higher concentrations of sugar alcohols and glutamic acid, whereas infants fed SF had higher concentrations of microbial polyphenol metabolites and amino acids than other feeding groups [107]. Studies focusing on differences between HM-fed and formula-fed infants have demonstrated similar results; however, no other study has explored the urinary metabolome of infants fed SF, apart from targeted approaches to measure various isoflavones [[109], [110], [111], [112], [113]]. Since urinary metabolites reflect general metabolism [107] and purines play important roles in the intermediary metabolism and cell signaling [43], these results suggest that early feeding mode may influence multiple metabolic pathways in infancy.

### Microbiome

HM-fed infants exhibited the lowest microbial α-diversity, the highest relative abundance of beneficial bacteria such as *Bifidobacterium* and *Bacteroides*, and the highest concentration of butyric acid compared with those fed MF or SF during the first year of life. In contrast, SF-fed infants presented the highest gut microbial α-diversity throughout the first year of life. Differences between SF-fed and MF-fed infants extended beyond α-diversity. The abundance of unidentified genera between the formula fed groups was observed through the ages: SMB53 was significantly lower in the MF than SF-fed infants at 3, 6, and 9 mo of age; *Ruminococcaceae* family was enriched in the SF-fed compared with that in MF-fed infants at 3 mo of age; and at 6 mo of age, *Lachnospiraceae* family and *Clostridiales*, *Coprococcus*, and *Roseburia* were higher in the SF-fed infants than in the MF-fed infants [69]. These findings demonstrate the associations between infant feeding and the developing microbiota through the first year of life.

Analysis of fecal samples collected over time from children showed that breastfeeding status plays an important role in shaping the early-life gut microbial composition [114]. Indeed, HM appears to have a pivotal role in shaping early-life gut microbiota composition through its dynamic constituents and the continuous exposure of the infant to milk microbiota via the gut–mammary axis [106]. Although the definition of healthy gut lacks universal consensus in the literature, key characteristics in breastfed infants include the following: high *Bifidobacterium* abundance [115], efficient HM oligosaccharides utilization [116], production of short-chain fatty acids [117], lower microbial α-diversity compared with formula-fed infants [118], as well as reduced antimicrobial resistance and virulence genes [119]. This microbial profile has been associated with immune modulation, metabolic programming, bone turnover [120], and neurodevelopment [121]. These favorable patterns observed in the HM-fed group have potential implications for long-term metabolic and immune outcomes.

### Neurodevelopmental outcomes

#### Language development and neurophysiological measures

Early neurophysiologic evidence from the Beginnings Study indicates that infant feeding mode is associated with subtle, time-dependent differences in brain function during the first year of life. HM-fed infants demonstrate more advanced neural processing of speech, characterized by faster processing speeds, greater syllables discrimination, and more robust responses in brain regions linked to language comprehension than formula-fed infants [[70], [71], [72], [73]]. These differences appear to reflect higher-order linguistic processing rather than basic auditory perception, which remains largely comparable across feeding groups [70,73,74,122]. In contrast, SF-fed infants exhibit patterns suggestive of delayed neural maturation, including reduced sensitivity to speech sounds and greater reliance on right hemisphere processing at 12 mo [75]. Yet, early differences in brain activity patterns may reflect temporary changes and a compensatory strategy, where the brain recruits broader regions to make up for reduced activity in a key area, in order to still be able to distinguish among speech sounds, syllables, and tones [75]. Despite these early distinctions, most differences in brain response patterns attenuate over time, and formula-fed groups (SF and MF) are largely similar to one another across multiple measures [73,74,122]. Consistent with these findings, developmental trajectories of brain oscillatory activity reveal age-related increases in higher-frequency power (β and γ bands) across all groups, but with feeding specific modulation. HM-fed infants, particularly boys, exhibit greater cortical activation in higher-frequency bands associated with early cognitive processing, including increased prefrontal engagement, than SF-fed infants [[76], [77], [78],^123^].

Notably, differences are primarily observed in β and γ frequencies, with minimal variation in lower-frequency bands (δ, θ, and α), while the development of brain electrical activity is generally similar between formula-fed groups, as reported in 2 studies that explored the impact of early infant feeding groups on age-related development of higher frequency signal power [76,78].

Overall, these findings suggest that while early diet may influence the timing and characteristics of neurodevelopment, particularly in domains related to language processing and cortical activation, these effects are modest and sex specific and tend to converge across groups beyond infancy. Further longitudinal research may determine if those neurodevelopmental variations influence later academic performance and behavioral outcomes.

#### Cognitive and motor development

All scores on developmental testing between at 3, 6, 9, and 12 mo of age were within established normal ranges, and MF- and SF-fed groups did not differ significantly [35]. During the first year of life, HM-fed infants showed a small but statistically significant advantage for cognitive development (mental and language) compared with formula-fed infants. Across the first 5 y of life, all standard scores were also within established norms for all feeding groups [79]. HM-fed children showed significantly higher scores for motor development, composite intelligence, verbal intelligence, and language development compared with formula-fed children, whereas MF and SF-fed children did not differ significantly from each other [79]. At age 6 y, there were no differences in language, memory, or intelligence scores between the 3 feeding groups [34]. There was a significant increase in rhythmicity (the predictable recurrence of the child’s response to everyday events) found between SF-fed and HM-fed children (+0.2 standard score for SF-fed children) between 4 and 6 y of age, although these differences were small and not clinically significant. There were no significant differences for other temperament subscales between feeding groups [35].

**FIGURE 3:**
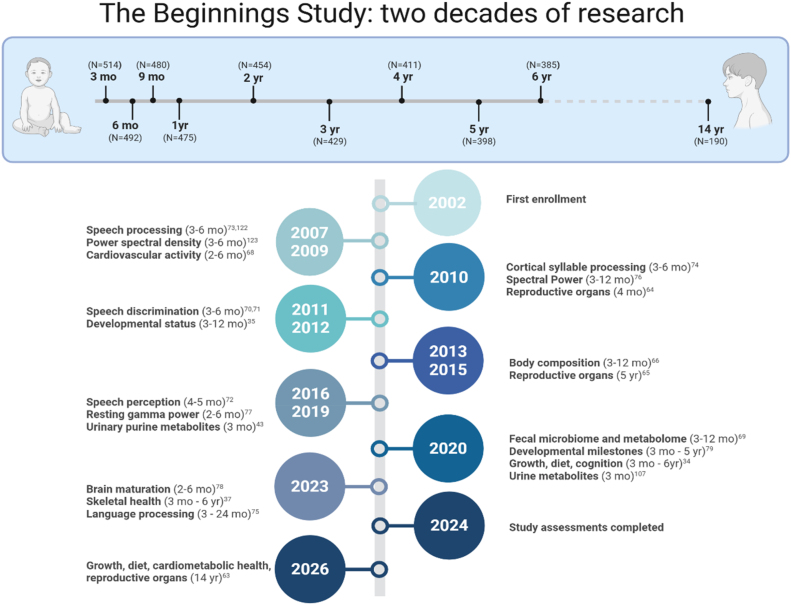
Research timeline of the Beginnings Study and Beginnings Follow-Up Study: 2 decades of research (2002–2026).

**FIGURE 4:**
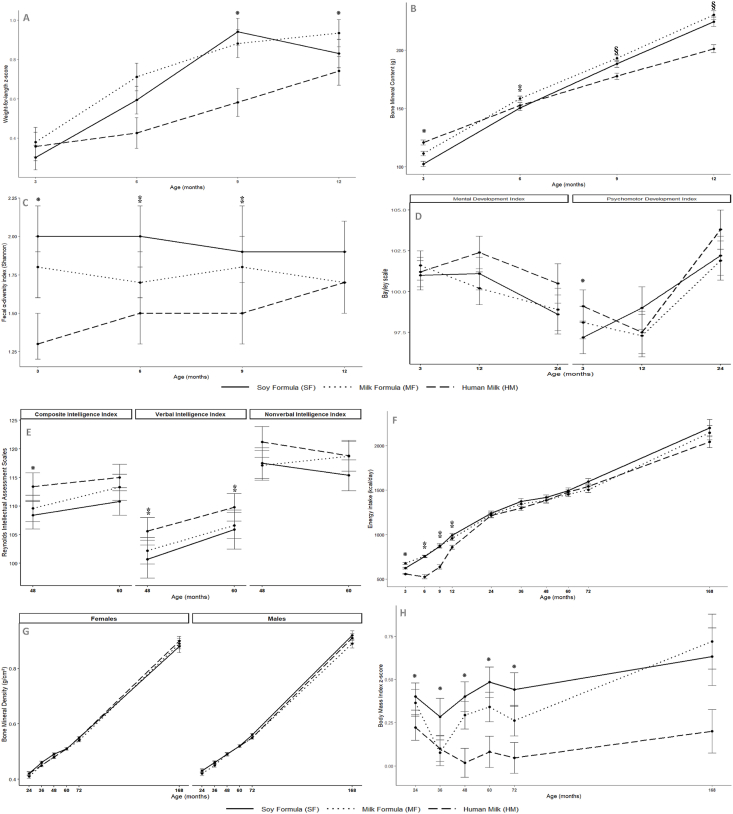
The Beginnings Study and Beginnings Follow-Up Study: key findings. Data are presented as estimated marginal means (SEM), adjusted for covariates described previously. (A) Weight-for-length *z*-score during the first year of life (3, 6, 9, and 12 mo), in participants fed SF, MF, and HM as infants. ∗SF = MF > HM. (B) Bone mineral content (g) for the first year of life (3, 6, 9, and 12 mo), in participants fed SF, MF, and HM as infants. ∗SF < MF < HM; ⁑SF < MF; §SF = MF > HM. (C) Fecal α-diversity index (Shannon), during the first year of life (3, 6, 9, and 12 mo), in participants fed SF, MF, and HM as infants. ∗SF > MF > HM; ⁑SF > MF = HM. (D) Bayley scales of infant development scores, from 3 to 24 mo of age, in participants fed SF, MF, and HM as infants. ∗SF< MF = HM. (E) Reynolds intellectual assessment scales composite score, at 48 and 60 mo of age, in participants fed SF, MF, and HM as infants. ∗SF = MF < HM; ⁑SF = MF, MF = HM, SF < HM. F. Energy intake (kcal/d), from 3 to 168 mo, in participants fed SF, MF, and HM as infants. ∗SF = MF, SF = HM, MF ≠ HM; ⁑SF = MF > HM. (G) Bone mineral density (g/cm^2^) (whole body without the head by DXA), from 24 to 168 mo, in female and male participants fed SF, MF, and HM as infants. (H) BMI *z*-score, from 24 to 168 mo, in participants fed SF, MF, and HM as infants. ∗SF = MF > HM. HM, human milk; MF, cow milk–based infant formula; SF, soy protein–based infant formula.

The neurodevelopmental benefits observed for breastfed children between 3 and 5 y of age were no longer present by age 6 y, suggesting that the early cognitive advantage may not persist long-term. Similar trends have been reported in large population-based cohort studies, including the UK Millenium Cohort Study [124] and Project Viva [125]. The UK Millenium Cohort Study is a nationally representative longitudinal study that assessed the association between breastfeeding and cognitive development at age 5 y, with a specific focus on children born preterm. Project Viva, a United States prospective prebirth cohort that followed up mothers and children from 1999 to 2002, also examined effects of breastfeeding duration and exclusivity on child cognition. Both studies reported moderate benefits of breastfeeding on early intelligence and noted these benefits were strongly explained by socioeconomic and parental confounders, mirroring the conclusion of a systematic review on this topic [[124], [125], [126]]. While HM is identified as the best option for infant nutrition, it is encouraging that formula-fed infants demonstrate comparable cognitive outcomes with HM-fed infants when breastfeeding is not feasible for families [127]. Planned analyses of intelligence and academic performance measures at age 14 y will help decipher whether long-term effect of infant feeding practices is present during adolescence. Although some studies [124,125] suggest that early cognitive advantages associated with breastfeeding may attenuate over time after adjustment for socioeconomic and parental factors, other studies [[128], [129], [130], [131]] have reported associations between breastfeeding and higher IQ and academic performance during adolescence. A summary of the main findings across all outcomes is presented in Table 1 and FIGURE 3, FIGURE 4.

**TABLE 1:** Main findings of the Beginnings Study and Beginnings Follow-Up Study through the years (2002–2026)^1^

Variable	Outcome	Age	Results	Reference
Dietary intake	Energy (kcal/d)	3 mo	SF < MF >HM	Sobik et al. [34], Leandro et al. [63]
		6–12 mo	SF = MF > HM	
	Protein (%kcal/d)	3, 6 mo	SF > MF > HM	
		9 mo	SF > MF = HM	
		12 mo	SF = MF, SF= HM	
Dietary intake	Carbohydrate (%kcal/d)	3, 12 mo	SF = MF = HM	Sobik et al. [34]
		6 mo	SF < MF = HM	
		9 mo	SF < MF < HM	
	Fat (%kcal/d)	3 mo	MF < SF < HM	
		6 mo	SF > MF = HM	
		9 mo	SF > MF > HM	
		12 mo	SF = MF = HM	
	Energy (kcal/d) and macronutrients (%kcal/d)	2–6 y	SF = MF = HM	Sobik et al. [34], Leandro et al. [63]
		14 y		
	Fat (%kcal/d)	14 y	SF = MF, SF = HM, HM < MF	
Body composition and growth	Weight gain velocity (g/d)	3, 12 mo	SF = MF > HM	Andres et al. [66]
	Weight for length *z*-score	3, 6 mo	SF = MF = HM	
		9, 12 mo	SF = MF > HM	
	FM (%)	3, 6 mo	SF = MF > HM	
	BMI-for-age *z*-score	2–6 y	SF = MF > HM	Sobik et al. [34], Leandro et al. [63]
		14 y	SF = MF = HM	
	FMI	3, 6 mo	SF = MF < HM	Sobik et al. [34]
		3, 4 y	SF = MF > HM	
		5, 6 y	SF = MF > HM	
	FMI and WC	14 y	SF = HM < MF	Leandro et al. [63]
Skeletal health	BMC (g/cm)	3 mo	SF < MF < HM	Andres et al. [66]
		6 mo	SF < MF^2^	
		9, 12 mo	SF = MF > HM	
	BMD	1–6 y	SF = MF = HM	Chen et al. [37]
	Urinary NTx, Osteocalcin	2, 5, 6 y	SF = MF = HM	
Cardiovascular health	HR	4 mo	HM > MF = SF	Pivik et al. [68]
		5–6 mo	SF = MF = HM	
	HRV	4, 6 mo	SF = MF = HM	
	VT rate of change (males)	9–24 mo	HM < MF = SF	Pivik et al. [67]
	VT stability	3–24 mo	SF < MF = HM	
Reproductive organs	Ovarian volumes	4 mo	MF > HM^2^	Gilchrist et al. [64]
	Testicular volume	4 mo	SF = MF < HM	
	Breasts, ovaries, testes, and uteri	5, 14 y	SF = MF = HM	Andres et al. [65], Leandro et al. [63]
Microbiota and metabolome	Polyphenols metabolites and amino acid (urine)	3 mo	SF > MF = HM	Rosa et al. [107]
Microbiota and metabolome	Purine metabolites (urine)	3 mo	SF > MF = HM	Aguilar-Lozano et al. [43]
	Gut microbiota diversity	3–9 mo	SF > HM^2^	Brink et al. [69]
	*Bifidobacterium* abundance	3–12 mo	SF < HM^2^	
	Butyric acid (feces)	3–9 mo	SF = MF < HM	
Language development	Sound content processing	3–6 mo	MF = HM^3^	Pivik et al. [122]
	Encoding speed	4–5 mo	SF = MF < HM	Pivik et al. [[70], [71], [72]]
	Syllable discrimination (P350)	4–5 mo	SF = MF = HM	Pivik et al. [72], Jing et al. [73], Li et al. [74]
	Phonological awareness	3–24 mo	SF < MF < HM	Alatorre-Cruz et al. [75]
	Brain maturity	12 mo	SF < HM < MF	Alatorre-Cruz et al. [75]
Neurophysiological measures	Overall power (males)	3–12 mo	SF = MF < HM	Jing et al. [76]
	Overall power (females)	3 mo	SF > MF = HM	
	β power	2, 6 mo	SF = MF < HM	Gilbreath et al. [78]
	γ power	2–6 mo	Females: SF = HM < MF	Pivik et al. [77]
		2–3 mo	Males: SF = HM < MF	
		4 mo	Males: HM > SF > MF	
		5–6 mo	Males: SF = MF < HM	
	Prefrontal activation	2, 6 mo	SF < MF < HM	Gilbreath et al. [78]
Temperament outcomes	Rhythmicity (Carey)	6 mo	HM > MF^2^	McCorkle et al. [62]
		4 y	SF > MF^2^	
		5–6 y	SF > HM^2^	
	Approach (Carey)	6–12 mo	SF = MF = HM	
		2 y	SF = MF > HM	
		3–6 y	SF = MF = HM	
	Adaptability (Carey)	6 mo	HM > MF^2^	
		0.75–6 y	SF = MF = HM	
	Intensity (Carey)	0.5–6 y	SF = MF = HM	
	Mood (Carey)	0.5–3 y	SF = MF = HM	
		4 y	SF > MF^2^	
		5, 6 y	SF = MF = HM	
Cognitive and motor development	Cognitive development (BSID-II)	3 mo	SF = MF = HM	Andres et al. [35], Bellando et al. [79]
		6 mo	SF < HM^2^	
		9, 12 mo	SF = MF < HM	
		24 mo	SF = MF = HM	
	Motor development (BSID-II)	3, 6 mo	SF < HM^2^	
		9, 12, 24 mo	SF = MF = HM	
	Composite intelligence (RIAS)	4, 5 y	SF = MF < HM	
	Language development (PLS-3)	3, 6 mo	SF = HM > MF	
		9, 12, 24 mo	SF = MF = HM	
		3–5 y	SF = MF < HM	
		6 y	SF = MF = HM	
	General memory index (CMS)	6 y	SF = MF = HM	Sobik et al. [34]
	Full-scale IQ (WPPSI)	6 y	SF = MF = HM	

1 Abbreviations: BMC, bone mineral content; BMD, bone mineral density; BSID-II, Bayley scales of infant development II; Carey, Carey temperament scales; CMS, Children’s Memory Scale; FMI, fat mass index; HM, human milk; HR, heart rate; HRV, heart rate variability; MF, cow milk–based infant formula; NTx, N-terminal telepeptide of type I collagen; PLS-3, preschool language scale-3; RIAS, Reynolds intellectual assessment scales; SF, soy protein–based infant formula; VT, vagal tone; WC, waist circumference; WPPSI, Wechsler Preschool and Primary Scale for Children, WASI-II, Wechsler Abbreviated Scale of Intelligence, second edition.

2 The omitted group presented intermediate value that did not differed significantly from either of the other 2 groups.

3 Only 2 feeding groups were compared.

### Strengths and Limitations

Some limitations of the study include the following: *1*) the absence of blood samples during the first 6 y of life that could serve as markers of dietary intake and measurement of bioactive compounds intake, such as polyphenols; *2*) the absence of 24-h movement behavior during the first 6 y of life; *3*) the absence of paternal health indicators; *4*) the low rate of retention for the Beginnings Follow-Up Study (14 y-visit: 31% of enrolled participants, 36% eligible participants, and 49.4% of the completers at age 6 y), which is consistent with other long-term pediatric cohort studies [[132], [133], [134]]; and *5*) an unbalanced follow-up at age 14 y with greater retention (*n* = 76) for the HM group than for the SF group (*n* = 52). All these limitations may introduce bias in the interpretation of the long-term outcomes, and the results should be interpreted within these contexts. Despite the limitations, the Beginnings Study presents several relevant strengths that enhance its scientific importance as follows: *1*) prospective longitudinal design, following participants from infancy through adolescence, which allows for the evaluation of both immediate- and long-term outcomes (up to 14 y old); *2*) broad spectrum of outcomes, including growth, body composition, skeletal health, cardiovascular function, gut microbiota profiles, urinary metabolites, neurodevelopmental testing, and reproductive maturation, facilitating an integrative understanding how nutrition can influence development through different stages of the life cycle; and *3*) consideration for important covariates. In addition, the inclusion of 3 distinct feeding groups provides a unique comparative design not common in large-scale pediatric cohorts. The rigorous, standardized data collection protocols and repeated measurements at multiple developmental stages reduced measurement bias. Additionally, the use of gold standard devices and analysis, including dual-energy x-ray absorptiometry, peripheral quantitative computed tomography, electroencephalogram, and microbiome profiling, provides a detailed understanding of the biological pathways connecting early nutrition with long-term health outcomes.

## Conclusions

While a recent review by Abrams et al. [135] examining SF concerns emphasizes that current international guidelines limit SF as a third-line infant feeding option [32,136] for those with IgE-mediated CMA, primarily due to assumptions of a potential for phytoestrogens exposure and crossreactivity, emerging evidence challenge these assumptions [137,138]. Most isoflavones in SF do not warrant classification as endocrine disruptors, as their intake has not been associated clinically relevant endocrine or reproductive abnormalities [139]. Moreover, large prospective studies demonstrate that coallergy between soy and cow milk is far less common than previously assumed [137,138,140]. Safety assessments consistently demonstrate no adverse effects of SF on growth, bone mineralization, cognitive development, or pubertal timing [141]. The Beginnings Study and Beginnings Follow-Up Study results highlighted in this review provide additional evidence of the absence of adverse effects on growth, body composition, skeletal health, cardiovascular function, gut microbiota profiles, urinary metabolites, neurodevelopment, and reproductive maturation. Collectively, these findings support the safety of modern SF when used under appropriate clinical guidance and reinforce the importance of optimizing infant nutrition policies, ensuring formula composition aligns with current scientific evidence and considers environmental and parental factors that influence infant long-term health.

## Author contributions

The authors’ responsibilities were as follows—AA and MBM: designed the research; AA, BJB, J-RC, TE, KT, LJL-P, MBM, CS, SS, and MW: conducted research; AA and JS: designed the structure of the manuscript; and all authors: wrote, read, and approved the final manuscript. AA had primary responsibility for final content.

## Declaration of Generative AI and AI-assisted technologies in the writing process

The authors declare that no generative AI or AI-assisted technologies were used in the writing of this manuscript.

## Funding

This study was supported by USDA Agricultural Research Service (grant/award number: 6026-10700-001-000D). The funders had no role in study design, data collection and analysis, or preparation of the manuscript. The supporting source had no such involvement or restrictions regarding publication.

## Conflict of interest

AA is an Editorial Board Member for *Advances in Nutrition* and played no role in the Journal’s evaluation of the manuscript.
