# Supplementary material for: Longitudinal Evaluation from Birth to Adolescence of Soy Protein–Based Infant Formula Compared with Cow Milk–Based Formula and Breastfeeding: A Comprehensive Summary of Findings
Source: Adv Nutr. 2026 Jun 6;17(7):100669. doi: 10.1016/j.advnut.2026.100669 (PMC13316684; doi:10.1016/j.advnut.2026.100669)
Supplement: Multimedia component 1 [file mmc1.docx]

**Supplemental Table S1:** Overview of covariates used in the *Beginnings Study* and *Beginnings Follow Up Study* analyses by outcome domain, 2002-2026.

| Outcome domain | Covariates | Age | Reference |
| --- | --- | --- | --- |
| Dietary intake | Child sex, race, gestational age, birth weight, birth length, and weeks strictly on feeding diet. | 3 -72 months | Sobik et al. (34) |
|  | Child age, sex, race/ethnicity, maternal education level, birth weight, and gestational age. | 14 years | Leandro et al. (63) |
| Body Composition & Growth | Child sex, race, gestational age, birth weight, birth length, feeding history, and maternal SES. | 3-12 months | Andres et al. (66) |
|  | Child sex, child race, gestational age, birth weight, birth length, and weeks strictly on feeding group. | 3 -72 months | Sobik et al. (34) |
|  | Child age, sex, race/ethnicity, maternal education level, birth weight, and gestational age. | 14 years | Leandro et al. (63) |
| Skeletal Health | Child sex, race, gestational age, birth weight, birth length, feeding history, and maternal SES. | 3-12 months | Andres et al. (66) |
|  | Child age, sex, race, gestational age, birth weight, birth length, and age of complementary food introduction | 3 -72 months | Chen et al. (37) |
| Cardiovascular Health | Child age and sex. | 3-24 months | Pivik et al. (67) |
| Reproductive Organs | Child race, gestational age, and birth weight. | 5 years | Andres et al. (65) |
|  | Child age, sex, race/ethnicity, gestational age, and birth weight. | 14 years | Leandro et al.(63) |
| Microbiota & metabolome | Urinary creatinine | 3 months | Aguilar-Lozano et al. (43),  Rosa et al. (107) |
| Language development | Gestational age, birth weight, birth length, maternal IQ, parental education level, and SES | 4-5 months | Pivik et al. (70,71,72) |
|  | Gestational age, time on postnatal diet, SES and maternal IQ. | 4-5 months | Pivik et al.(72), Jing et al. (73), Li et al. (74) |
|  | Gestational age, maternal cognitive and psychiatric status, child sex, and PLS-3 scores. | 3-24 months | Alatorre-Cruz et al.(75) |
| Neurophysiological Measures | Child sex, and age. | 3-12 months | Jing et al. (76) |
|  | Child sex, gestational age, birth weight, maternal IQ, and head circumference. | 2, 6 months | Gilbreath et al.(78) |
|  | Gestational age, birth weight, onset diet group, child weight, head circumference, maternal SES | 2-6 months | Pivik et al. (77) |
| Temperament Outcomes | Child sex, gestational age, maternal IQ, parent’s education level, race, and income | 6 months – 6 years | McCorkle (62) |
| Cognitive  and Motor development | Child age, sex, race, gestational age, birth weight, Head circumference, diet history, mother’s SES, mother’s IQ, mother’s age. | 3 -12 months | Andres et al. (35) |
|  | Gestational age, child sex, race, parental education level, maternal IQ and cohesion score. | 3 months - 6 years | Bellando et al.(79) |
|  | Child sex, race, birth weight, weeks strictly on feeding group. | 6 years | Sobik et al. (34) |

**Abbreviations:** BMI=Body Mass Index; HEI= Healthy Eating Index; IQ = intelligence quotient; PLS-3= preschool language scale-3, SES = Socio-Economic Status (measured by Four-Factor Index of Social Position(s) (61).
