# Supplementary material for: Longitudinal Evaluation from Birth to Adolescence of Soy Protein–Based Infant Formula Compared with Cow Milk–Based Formula and Breastfeeding: A Comprehensive Summary of Findings
Source: Adv Nutr. 2026 Jun 6;17(7):100669. doi: 10.1016/j.advnut.2026.100669 (PMC13316684; doi:10.1016/j.advnut.2026.100669)
Supplement: Multimedia component 2 [file mmc2.docx]

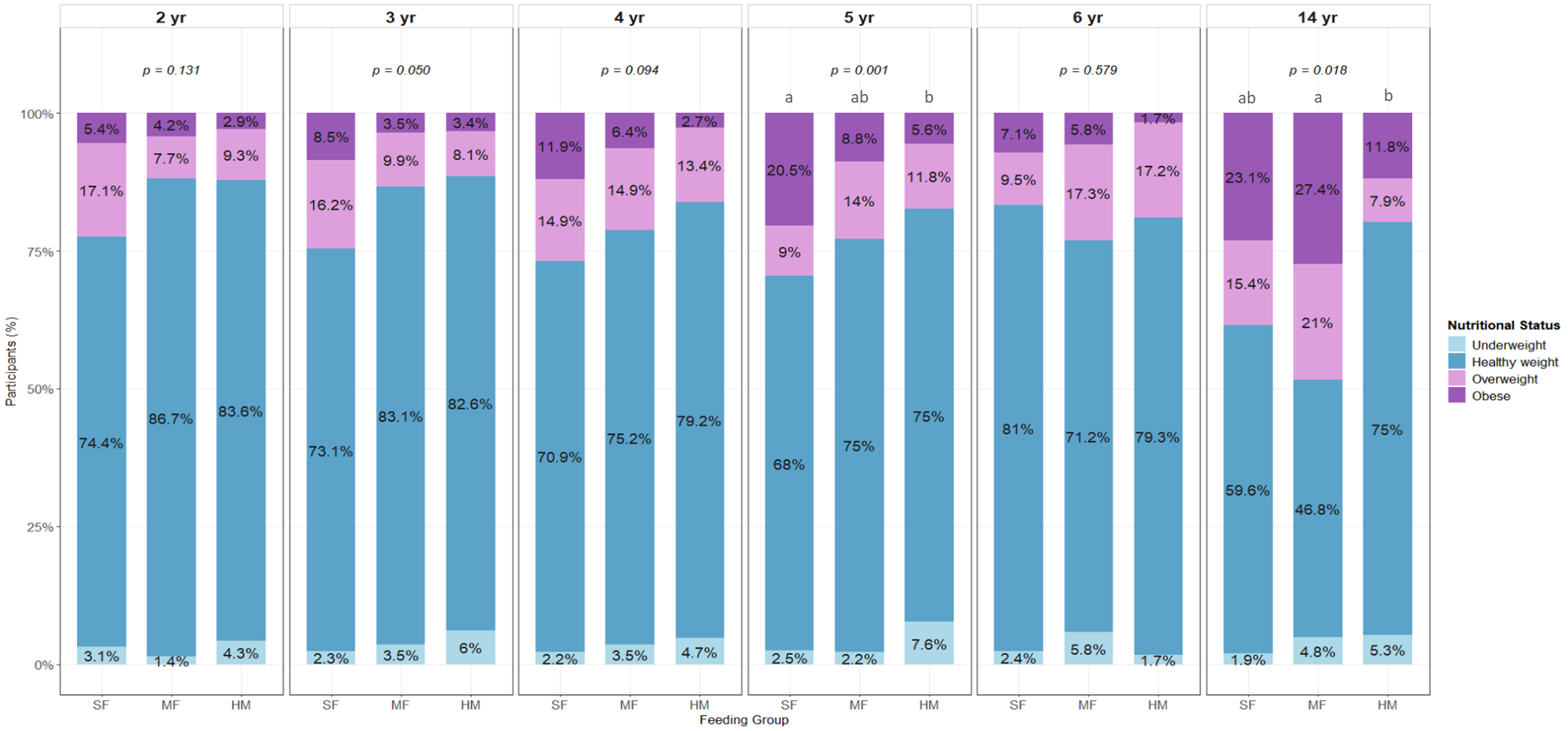


**Supplemental Figure S1:** Percent of participants with underweight, healthy weight, overweight or obesity from age 2 years to 14 years in the Beginnings Study and Beginnings Follow-Up Study.

**Abbreviations: HM:** human milk-fed children; **MF**: Cow’s milk-based infant formula-fed children; **SF:** Soy-based infant formula-fed children. Groups with differing letters are statistically different from one another.
